# Lake size and fish diversity determine resource use and trophic position of a top predator in high-latitude lakes

**DOI:** 10.1002/ece3.1464

**Published:** 2015-03-23

**Authors:** Antti P Eloranta, Kimmo K Kahilainen, Per-Arne Amundsen, Rune Knudsen, Chris Harrod, Roger I Jones

**Affiliations:** 1 Aquatic Ecology Department, Norwegian Institute for Nature ResearchP.O. Box 5685 Sluppen, NO-7485, Trondheim, Norway; 2 University of Jyväskylä, Department of Biological and Environmental SciencesP.O. Box 35, FIN-40014, Jyväskylä, Finland; 3 Department of Environmental Sciences, University of HelsinkiP.O. Box 65, FIN-00014, Helsinki, Finland; 4 Kilpisjärvi Biological Station, University of HelsinkiKäsivarrentie 14622, FIN-99490, Kilpisjärvi, Finland; 5 Department of Arctic and Marine Biology, UiT The Arctic University of NorwayP.O. Box 6050 Langnes, NO-9037, Tromsø, Norway; 6 Universidad de Antofagasta, Instituto de Ciencias Naturales Alexander von HumboldtAvenida Angamos 601, Antofagasta, Chile

**Keywords:** Benthic, energy mobilization, food-chain length, habitat coupling, lake morphometry, predation, resource competition, stable isotope analysis, trophic niche

## Abstract

Prey preference of top predators and energy flow across habitat boundaries are of fundamental importance for structure and function of aquatic and terrestrial ecosystems, as they may have strong effects on production, species diversity, and food-web stability. In lakes, littoral and pelagic food-web compartments are typically coupled and controlled by generalist fish top predators. However, the extent and determinants of such coupling remains a topical area of ecological research and is largely unknown in oligotrophic high-latitude lakes. We analyzed food-web structure and resource use by a generalist top predator, the Arctic charr *Salvelinus alpinus* (L.), in 17 oligotrophic subarctic lakes covering a marked gradient in size (0.5–1084 km^2^) and fish species richness (2–13 species). We expected top predators to shift from littoral to pelagic energy sources with increasing lake size, as the availability of pelagic prey resources and the competition for littoral prey are both likely to be higher in large lakes with multispecies fish communities. We also expected top predators to occupy a higher trophic position in lakes with greater fish species richness due to potential substitution of intermediate consumers (prey fish) and increased piscivory by top predators. Based on stable carbon and nitrogen isotope analyses, the mean reliance of Arctic charr on littoral energy sources showed a significant negative relationship with lake surface area, whereas the mean trophic position of Arctic charr, reflecting the lake food-chain length, increased with fish species richness. These results were supported by stomach contents data demonstrating a shift of Arctic charr from an invertebrate-dominated diet to piscivory on pelagic fish. Our study highlights that, because they determine the main energy source (littoral vs. pelagic) and the trophic position of generalist top predators, ecosystem size and fish diversity are particularly important factors influencing function and structure of food webs in high-latitude lakes.

## Introduction

Ecological research has increasingly recognized the fundamental importance of habitat linkages to the structure and function of aquatic and terrestrial ecosystems (Polis et al. 1997; Vadeboncoeur et al. 2002; Marcarelli et al. 2011). For instance, terrestrial predators can use terrestrial, marine and/or freshwater prey depending on seasonal and spatial availability of different resources (Helfield and Naiman 2006; Killengreen et al. 2011; Middleton et al. 2013). Correspondingly, generalist fish top predators in lakes can use both littoral (benthic) and pelagic food resources and thereby link these different habitats and food-web compartments (Schindler and Scheuerell 2002; Vander Zanden and Vadeboncoeur 2002; Eloranta et al. 2013a). Such cross-habitat linkages by top predators have been shown to influence production, community structure, and food-web stability (Polis et al. 1997; Vadeboncoeur et al. 2005; Rooney and McCann 2012). Previous studies have found conflicting effects of ecosystem size, productivity, and disturbance on food-chain length (Takimoto and Post 2013; Warfe et al. 2013). However, in most studies, the identity of the top predator has changed across these gradients. Arctic charr (*Salvelinus alpinus* (L.)) is a circumpolar, generalist fish species characteristic of high-latitude lakes, and is a species that has exceptional niche plasticity (Klemetsen et al. 2003; Woods et al. 2013). Thus, high-latitude lakes containing Arctic charr offer an outstanding opportunity to study effects of ecosystem size, productivity, and disturbance on food-web structure and energy flow patterns in ecosystems with the same apex predator.

Food webs and autochthonous production in lake ecosystems are predominantly based on photosynthesis by pelagic phytoplankton and littoral benthic algae (e.g., Schindler and Scheuerell 2002; Solomon et al. 2011; Althouse et al. 2014). The relative importance of pelagic and littoral production to whole-lake primary and secondary production typically depends on lake morphometry, trophic status and water color (Vadeboncoeur et al. 2003, 2008; Althouse et al. 2014). In oligotrophic, clear-water high-latitude lakes, most primary and secondary production typically occurs in the littoral habitats and food-web compartments (Sierszen et al. 2003; Vadeboncoeur et al. 2003; Karlsson and Byström 2005; Ask et al. 2009). Recent stable isotope studies from oligotrophic subarctic lakes have demonstrated that littoral production can be the main energy source for generalist fish consumers throughout the year, despite high seasonal fluctuations in light, temperature, and food availability (Eloranta et al. 2013b; Hayden et al. 2014b). Although of great importance in highlighting the role of littoral primary and secondary production in high-latitude lakes, previous case studies have mainly been conducted in small lakes with simple fish communities and food-web structures.

Recent ecological research has argued that ecosystem size and spatial heterogeneity within ecosystems can largely determine the relative contributions of basal resources from different habitats to higher order consumers (Thompson and Townsend 2005; Dolson et al. 2009; Tunney et al. 2012). Case studies from subarctic lakes have suggested that lake depth and fish community structure are important factors determining the outcomes of trophic interactions and energy flow (e.g., Eloranta et al. 2013a; Hayden et al. 2013, 2014a). However, larger-scale studies relating resource use by a common top predator to lake abiotic and biotic characteristics, such as lake morphometry, productivity, and fish community structure, are lacking in species-poor, oligotrophic high-latitude lakes. Here, we consider how lake abiotic characteristics and fish community composition ultimately affect the littoral and pelagic resource use by a common circumpolar generalist top predator. We include a broad range of lake sizes to extend inferences drawn from previous studies of small lakes (Karlsson and Byström 2005).

Several factors may affect the littoral and pelagic resource use by generalist fish top predators in high-latitude lakes. For instance, eutrophication or increased humus concentration in the water, both promoted by climate change, can significantly affect light penetration and lead to reduced littoral primary production and food resources available to higher trophic levels (Vadeboncoeur et al. 2003; Karlsson et al. 2009) and further reduce fish production (Finstad et al. 2014). However, in oligotrophic, clear-water lakes, other abiotic (e.g., lake surface area, depth and altitude) and biotic factors (e.g., competitive and predatory interactions) probably play a major role in determining the predominant energy flow pathway to and the trophic positions of fish (Dolson et al. 2009; Woods et al. 2013; Hayden et al. 2014a). Altitude strongly influences water temperature and ice-cover period in high-latitude lakes, which potentially shape competitive interactions (Helland et al. 2011) and niche use by top predators (Tunney et al. 2014). Lake morphometry fundamentally influences several physical, chemical, and biological processes including stratification, productivity, and carbon and nutrient dynamics, as well as niche availability for benthic and pelagic invertebrate and fish species (Wetzel 2001). Unlike more frequently studied small and shallow high-latitude lakes, fish top predators in larger and deeper lakes may rely more on pelagic phytoplankton-based carbon due to the expected proportional reduction in littoral area and increase in pelagic prey resources.

In high-latitude lakes where several fish species coexist, resource competition and predatory interactions may be the main factors influencing resource use by fish top predators. For instance, brown trout *Salmo trutta* L. can restrict the niche of sympatric Arctic charr *Salvelinus alpinus* (L.) by dominating the littoral habitat and food resources as well as preying on small Arctic charr (L'Abée-Lund et al. 1993; Eloranta et al. 2013a). In some large high-latitude lakes with multispecies fish communities, the presence of small planktivorous prey fishes, together with strong competition for littoral resources, may promote the use of the pelagic niche by fish top predators (Kahilainen and Lehtonen 2003; Eloranta et al. 2015). Despite the fundamental importance of littoral–pelagic coupling and foraging by top predators on the structure and function of lake ecosystems (Schindler and Scheuerell 2002; Rooney and McCann 2012; Hayden et al. 2014b), no empirical studies have used extensive lake morphometry, productivity, and fish species richness gradients to test how littoral reliance and trophic position of fish top predators differ between oligotrophic high-latitude lakes with contrasting abiotic and biotic characteristics.

Besides affecting the relative importance of littoral and pelagic trophic pathways, lake size may also influence fish species diversity (Barbour and Brown 1974; Nolby et al. 2015) and food-chain length in lakes (e.g., Post et al. 2000; Takimoto and Post 2013). Increased fish species diversity may lead to substitution of intermediate consumers, but also to increased competition and predation, all of which may induce niche shifts by top predators (cf. Vander Zanden et al. 1999a,b). Hence, lake size and fish species richness are likely to have strong and complex influences on ecosystem functioning and energy flow pathways. Understanding such large-scale patterns in food-web structures is crucial for predicting potential effects of, for example, species invasions on the functioning of high-latitude lake ecosystems which have low biodiversity and are considered particularly susceptible to environmental changes (Schindler and Smol 2006).

Here, we used stable isotope and stomach contents analyses to examine food-web structure and, in particular, to estimate the trophic position and relative importance of littoral and pelagic energy sources to the long-term diet of top predators in 17 subarctic lakes across northern Fennoscandia. While stable carbon and nitrogen isotopes can provide valuable information about the predominant energy source (littoral vs. pelagic) supporting top predators and about food-chain length in lakes (Post et al. 2000), stomach contents analysis gives complementary information about the most recently ingested prey items with a high taxonomic resolution (Layman et al. 2012, and references therein). Our study lakes cover a marked gradient in size (area 0.5–1084 km^2^), depth (*Z*_max_ 12–95 m), altitude (12–679 m a.s.l.), and fish species richness (2–13 species) and thus provide an excellent opportunity for investigating large-scale patterns in the energy flow to top predators. We hypothesized that the expected proportional reduction in littoral area and increased resource competition from higher number of littoral fish species would induce Arctic charr to shift from the utilization of littoral to more pelagic food resources with increasing lake size. We also hypothesized that strong interspecific resource competition and the increased availability of energetically profitable planktivorous prey fishes in multispecies fish communities would promote a shift by Arctic charr to a higher trophic position, indicating increased food-chain length.

## Materials and Methods

### Lake characteristics

All 17 study lakes are dimictic, oligotrophic, or slightly mesotrophic lakes covering the main distribution area of Arctic charr in northern Finland and Norway (Table S1; Fig. S1, Supporting Information). The lakes are surrounded by birch *Betula* spp. or pine *Pinus sylvestris* L. forests and small patches of farmland, except for Saanajärvi and Gæsjavri which are situated above the tree line. The abiotic parameters measured from each lake, and finally used in our set of linear models, included surface area, relative depth (*Z*_r_; calculated following Wetzel 2001), altitude, nutrients (total nitrogen, total phosphorus), and Secchi depth. As we lacked data for mean depth from some lakes, we included relative depth as a proxy for bathymetry. Secchi depth was included as a proxy for water color and turbidity, which can both affect primary and secondary production in nutrient-poor lakes (Vadeboncoeur et al. 2008; Karlsson et al. 2009; Finstad et al. 2014). Altitude was included as a proxy for climate and temperature, which can affect production and niche use of top predators (Tunney et al. 2014). Water nutrient data were also included despite the rather similar low trophic states of the study lakes (Table S1, Supporting Information). The abiotic lake parameters were measured during field work or obtained from public databases and electronic maps maintained by Finnish (Lapland Centre for Economic Development, Transport and Environment, and National Land Survey of Finland) and Norwegian (Norwegian Water Resources and Energy Directorate) environmental administrations.

A total of 16 fish species have been recorded from the 17 study lakes (Table S1, Supporting Information). The smallest lakes are mainly inhabited by Arctic charr and a few brown trout, whereas in larger lakes, Arctic charr coexist with brown trout and three-spined stickleback *Gasterosteus aculeatus* L. or with whitefish *Coregonus lavaretus* (L.), grayling *Thymallus thymallus* (L.), burbot *Lota lota* (L.), and a few other fish species. In addition to the aforementioned fish species, perch *Perca fluviatilis* L., pike *Esox lucius* L., nine-spined stickleback *Pungitius pungitius* (L.), and minnow *Phoxinus phoxinus* (L.) are also present in the largest study lakes. Most fish species are considered native, but vendace *Coregonus albula* (L.), landlocked salmon *Salmo salar* m. sebago, lake trout *Salvelinus namaycush* Walbaum, and common bullhead *Cottus gobio* L. are known to have been introduced to some of the large Finnish study lakes. In some of the study lakes, whitefish has evolved into littoral, pelagic, and profundal morphs showing distinct trophic niches and morphologies (Harrod et al. 2010). Arctic charr occur as monomorphic populations except in Fjellfrøsvatn where two Arctic charr morphs (littoral normal and profundal dwarf) have been found to coexist (Amundsen et al. 2008). However, all profundal Arctic charr morphs were excluded from this study.

### Data collection and analysis

All samples for stable isotope (SIA) and stomach contents (SCA) analyses were collected between August and October in 2005–2010. The sampling of fish muscle tissue for SIA was performed in the late open-water season to examine the main food sources assimilated during the main growth period (Perga and Gerdeaux 2005; Eloranta et al. 2010; Hayden et al. 2014b). Fish were sampled from the littoral, pelagic, and profundal habitats using series of multimesh and standard gill nets (1.5–5.0 m high and 30–65 m long) with knot-to-knot mesh sizes ranging from 5 to 60 mm (details in Kahilainen and Lehtonen 2003; Eloranta et al. 2013a). In each lake, the gill net series were set overnight for a total of 3–10 nights. All fish captured were identified to species, measured (fork length, mm) and weighed (g) in the field laboratory. From Arctic charr, only individuals of fork length ≥150 mm were chosen for the subsequent SIA and SCA analyses, because Arctic charr of this size are more likely to be top predators and typically are subjected to reduced predation risk and thus potentially display more generalist habitat and diet use than the smaller conspecifics (L'Abée-Lund et al. 1993). Altogether, 895 and 1174 Arctic charr of fork length ≥150 mm were analyzed for SIA and SCA, respectively (Table S2).

For SIA, a small sample of dorsal muscle tissue was dissected from random subsamples of fish and stored at −20°C. Whenever gill net catches permitted, almost equal numbers of individuals were included from each habitat type to make the subsamples representative of the whole fish population. Qualitative samples of putative littoral and pelagic food sources were collected from each study lake for SIA. Zooplankton were collected from the pelagic zone by taking several hauls through the water column with a 50- to 100-*μ*m mesh plankton net until sufficient material was obtained. The samples were later sieved through a 200-*μ*m mesh to obtain pure samples of adult cladocerans and copepods. Benthic macro-invertebrates were collected from the littoral zone using a kick net in shallow water and an Ekman grab or a benthic sledge in deeper areas. All benthic samples were sieved through a 500-*μ*m mesh. Both benthic and pelagic invertebrates were sorted to genus level. Only the soft body tissue from mollusks was prepared for SIA.

Samples from fish, benthic macro-invertebrates, and zooplankton were dried (48 h in a freeze-drier or at 60°C in an oven), ground to a fine powder, and precisely weighed (0.5–0.6 mg) for subsequent SIA. Stable carbon and nitrogen isotope ratios (expressed as *δ*^13^C and *δ*^15^N, respectively) were analyzed by an elemental analyser coupled to a continuous flow isotope ratio mass spectrometer. Vienna PeeDee Belemnite and atmospheric nitrogen were used as international references for carbon and nitrogen, respectively. Standard deviation of an internal working standard was less than 0.3 ‰ for *δ*^13^C and 0.2 ‰ for *δ*^15^N. The fish muscle *δ*^13^C values were not corrected for lipids due to the generally low C:N ratios indicating negligible lipid content in samples.

The SIAR (Stable Isotope Analysis in R; Parnell et al. 2010) Bayesian isotopic mixing model was used to estimate the mean littoral reliance (LR_charr_), and the two-source isotopic mixing model of Karlsson and Byström (2005) was used to calculate the mean trophic position (TP_charr_) of Arctic charr in each study lake. For both models, only those benthic macro-invertebrates (mainly snails, amphipods, and chironomid larvae) sampled from the shallow (0–5 m depth) littoral zone and observed in Arctic charr stomach contents were chosen to calculate the littoral isotopic baselines (mean ± SD of *δ*^13^C and *δ*^15^N), whereas all zooplankton samples (cladocerans and copepods) were pooled for the pelagic baselines. The commonly assumed fractionation factors of 0.4 ± 1.3 ‰ for *δ*^13^C and 3.4 ± 1.0 ‰ for *δ*^15^N (Post 2002) were used in the models. Concentrations (mean ± SD %) of C and N in the littoral and pelagic food sources were also incorporated into the SIAR model. Despite their different statistical approaches, we found the mean LR_charr_ estimates calculated using SIAR and using the linear two-source mixing model of Karlsson and Byström (2005) to be consistent (paired *t*-test: *t *= −0.074, df* *= 16, *P *=* *0.942).

Random subsamples of Arctic charr were chosen for SCA to study taxonomic composition of prey items and to complement the isotopic estimates. The total stomach fullness was determined visually on a percentage scale ranging from empty (0%) to full (100%), and the relative contribution of each prey taxon to the total stomach contents was estimated according to Amundsen et al. (1996). The relative contributions of (1) benthic macro-invertebrates (insect larvae, mollusks, benthic crustaceans, and adult and pupal stages of aquatic insects); (2) pelagic crustaceans (cladocerans, copepods, and *Mysis* spp.); and (3) fish in the stomach contents were finally calculated for each Arctic charr population.

Finally, several linear models were compared to study how LR_charr_ and TP_charr_ were related to lake abiotic characteristics and fish species richness (Table S1). Model selection was performed by stepwise removal of terms to minimize AIC, using aictab function in AICcmodavg package (Mazerolle 2015). The simplest model with fewest terms was chosen when two models were equally supported (ΔAIC < 2). The full models were of the form:

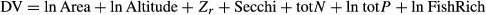
where DV represents the dependent variable (LR_charr_ or TP_charr_), *Z*_r_ relative depth, Secchi Secchi depth, tot*N* and tot*P* total nitrogen and phosphorus, and FishRich fish species richness. Lake surface area, altitude, total phosphorus, fish species richness, and trophic position of Arctic charr were ln-transformed to normalize the data. The normality of model residuals was tested using Shapiro–Wilk test. All statistical analyses were performed in R 3.1.2 (R Core Team 2014).

## Results

### Community structure

The relative proportion of Arctic charr in the total fish catch differed between the study lakes (Table S1). Arctic charr was the dominant fish species in lakes where it coexisted with just 1–2 other fish species. In contrast, in multispecies fish communities, Arctic charr made only a small contribution to the total fish catch, and whitefish was numerically the dominant fish species, particularly in the largest study lakes. Arctic charr also showed marked differences in size distributions between the study lakes, with the mean fork length ranging from 187 mm to 432 mm (Table S2).

**Figure 1:**
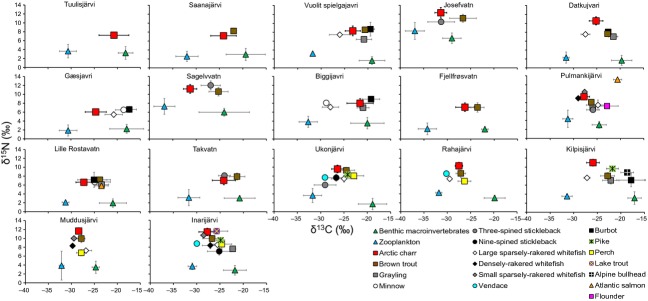
Stable isotope biplots representing *δ*^13^C and *δ*^15^N values (mean ± SD) of littoral and pelagic primary consumers and of different fish species. The lakes are arranged from left to right by increasing surface area.

Typical zooplankton taxa collected from the study lakes included cladocerans (*Daphnia* spp., *Bosmina* spp. and *Holopedium gibberum* Zaddach) and calanoid copepods (*Eudiaptomus graciloides* Liljeborg). The most common littoral benthic macro-invertebrate taxa included chironomid larvae, the amphipod *Gammarus lacustris* Sars, trichopteran larvae, the gastropod *Lymnaea* sp., and the bivalve *Pisidium* sp. The *δ*^13^C and *δ*^15^N values indicated clear isotopic separation between the littoral and pelagic consumers and between different trophic levels, respectively (Fig.1). Littoral benthic macro-invertebrate *δ*^13^C values were on average 6.7–14.3 ‰ higher than those of zooplankton, whereas mean *δ*^15^N values differed by only 0.09–1.9 ‰. Arctic charr mean *δ*^13^C and *δ*^15^N values showed marked differences between the lakes (Fig.1). Arctic charr mean *δ*^13^C values were generally lower (i.e., more pelagic) than those of sympatric littoral-dwelling fish species such as brown trout, grayling, burbot, pike, and perch, but higher (i.e., more littoral) than those of specialist pelagic planktivorous fish species such as whitefish and vendace. The Arctic charr mean (±SD) *δ*^15^N values were on average 5.8 ‰ (±1.6) higher than littoral and pelagic baselines, with the difference ranging from 3.8 to 8.6 ‰ (notionally equivalent to 1.1–2.5 trophic levels) among the study lakes (Fig.1).

### Trophic niche of Arctic charr

**Figure 2:**
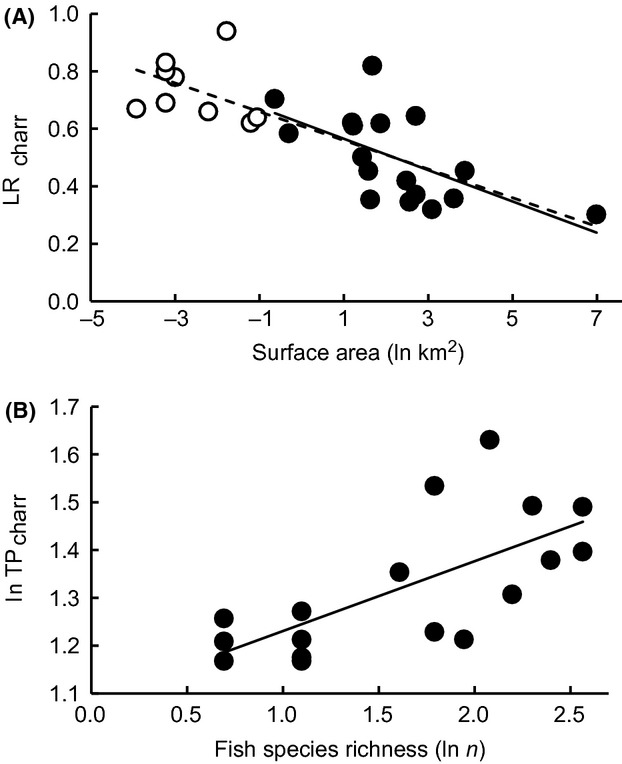
Relationships between (A) mean littoral reliance of Arctic charr (LR_charr_) and lake surface area (ln km^2^) and (B) mean trophic position of Arctic charr (TP_charr_) and fish species richness (ln *n*). Lake area, fish species richness and TP_charr_ were ln-transformed to normalize the data. The dashed line in (A) indicates the relationship between mean LR_charr_ and lake area based on the present data (solid symbols) and the data presented by Karlsson and Byström (2005) from nine small subarctic lakes (open symbols). See Tables1 and 2 for details of model selection and parameter estimates, respectively.

When averaging SIAR estimates of littoral reliance (LR_charr_) across all study lakes, Arctic charr relied equally (50/50%) on littoral and pelagic carbon sources; however, there were clear between-lake differences with mean LR_charr_ ranging between 30% and 82% (Table S2). The results from linear models indicated a significant negative relationship between LR_charr_ and lake surface area (Tables1 and 2, Fig.2A). Inclusion of other explanatory variables did not significantly improve the model (Tables1 and 2). To supplement the present data and to test for the reliability of the final model, we repeated the modeling after including the SIA and lake abiotic data presented by Karlsson and Byström (2005) from nine small and shallow Swedish subarctic lakes. Including these data into the model produced the same result and strengthened the observed negative trend between LR_charr_ and lake surface area (Tables1 and 2, Fig.2A). The model including fish species richness indicated a slight negative trend between LR_charr_ and fish species richness and was equally parsimonious (ΔAIC < 2) as the model with only lake area as a predictor.

**Table 1:** Results for model selection for (a–b) littoral reliance (LR_charr_) and (c) trophic position (TP_charr_) of Arctic charr modeled with lake abiotic parameters and fish species richness as explanatory variables. Number of estimated parameters for each model (K), AIC, difference in AIC (AIC_i_–AIC_min_) and Akaike weights (W_i_) for candidate models are shown. For data normalization, lake area, total phosphorus, fish species richness, and the mean trophic level of Arctic charr were ln-transformed. Lowest AIC values indicate the best (most parsimonious) models predicting LR_charr_ and TP_charr_. LR_charr_ is modeled both (a) using the stable isotope and lake data in this study and (b) by including the data from Karlsson and Byström (2005) study (lacks Secchi depth data)

Model	K	AIC	ΔAIC	Wi
(a)				
ln Area	3	−16.62	0.00	0.72
ln Area + tot*N*	4	−14.10	2.52	0.20
ln Area + tot*N* + ln tot*P*	5	−11.47	5.15	0.05
ln Area + tot*N* + ln tot*P* + ln Altitude	6	−9.29	7.33	0.02
ln Area + tot*N* + ln tot*P* + ln Altitude + ln FishRich	7	−4.90	11.72	0.00
ln Area + tot*N* + ln tot*P* + ln Altitude + ln FishRich + Secchi	8	0.02	16.64	0.00
ln Area + tot*N* + ln tot*P* + ln Altitude + ln FishRich + Secchi + *Z*_*r*_	9	9.23	25.85	0.00
(b)				
ln Area	3	−32.54	0.00	0.55
ln Area + ln FishRich	4	−31.37	1.17	0.31
ln Area + ln FishRich + ln Altitude	5	−29.35	3.19	0.11
ln Area + ln FishRich + ln Altitude + ln tot*P*	6	−26.29	6.26	0.02
ln Area + ln FishRich + ln Altitude + ln tot*P* + tot*N*	7	−22.90	9.64	0.00
ln Area + ln FishRich + ln Altitude + ln tot*P* + tot*N* + *Z*_*r*_	8	−18.70	13.84	0.00
(c)				
ln FishRich	3	26.25	0.00	0.44
ln FishRich + *Z*_*r*_	4	26.29	0.04	0.43
ln FishRich + *Z*_*r*_ + Secchi	5	28.84	2.60	0.12
ln FishRich + *Z*_*r*_ + Secchi + tot*N*	6	33.18	6.93	0.01
ln FishRich + *Z*_*r*_ + Secchi + tot*N* + ln tot*P*	7	37.80	11.56	0.00
ln FishRich + *Z*_*r*_ + Secchi + tot*N* + ln tot*P* + ln Area	8	44.95	18.71	0.00
ln FishRich + *Z*_*r*_ + Secchi + tot*N* + ln tot*P* + ln Area + ln Altitude	9	54.51	28.26	0.00

**Table 2:** Parameter estimates and corresponding *t*- and *P*-values for the final selected models with (a–b) littoral reliance (LR_charr_) and (c) trophic position (TP_charr_) of Arctic charr as response variables and lake area and fish species richness as predictor variables (both ln-transformed for data normalization)

	Parameter (±SE)	*t*-value	*P*
(a)			
Intercept	0.62 (±0.05)	12.20	<0.001
ln Area	−0.05 (±0.02)	−2.97	0.010
(b)			
Intercept	0.61 (±0.02)	25.84	<0.001
ln Area	−0.05 (±0.01)	−5.84	<0.001
(c)			
Intercept	2.88 (±0.29)	9.95	<0.001
ln FishRich	0.56 (±0.16)	3.42	0.004

Arctic charr *δ*^15^N values suggested that the species typically represented the top predator in our samples collected from the study lakes (Fig.1), with a mean (±SD) trophic level calculated from the *δ*^15^N values of 3.8 (±0.6). However, TP_charr_ differed markedly between the study lakes (range: 3.2–5.1; Table S2). The results from linear models indicated a significant positive relationship between TP_charr_ and fish species richness (Table1 and 2, Fig.2B). The model including lake relative depth indicated a slight negative trend between TP_charr_ and lake relative depth and was equally parsimonious (ΔAIC < 2) as the model with only fish species richness as a predictor.

**Figure 3:**
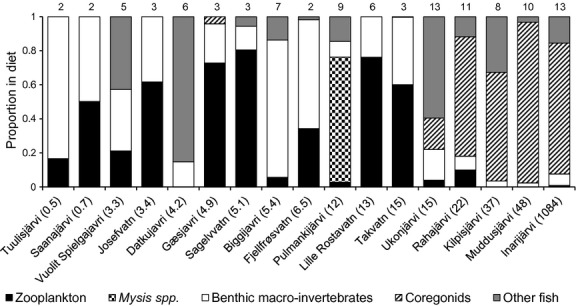
Relative proportion of different prey items in Arctic charr stomach contents. Lakes are arranged from left to right by increasing surface area (shown in parentheses, km^2^), and number of fish species present in each lake is shown above the bars.

The observed patterns in Arctic charr littoral reliance and trophic position with increasing lake size and fish species richness were further supported by the SCA data (Fig.3). The relative proportion of benthic macro-invertebrates in Arctic charr stomach contents decreased with increasing lake surface area, whereas the dietary proportion of fish increased with fish species richness (Fig. S2A–B). Benthic *G. lacustris* amphipods and *Lymnaea* sp. snails were particularly important benthic prey for Arctic charr in small lakes where the species coexisted with brown trout (i.e., Tuulisjärvi and Saanajärvi). The observed negative trend in Arctic charr benthivory was associated with increased planktivory (particularly on *Daphnia* spp. and *Bosmina* spp. cladocerans) in medium-sized lakes and piscivorous predation on planktivorous coregonids (whitefish and vendace) in large lakes with multispecies fish communities (Fig.3). The predatory cladocerans *Bythotrephes longimanus* Leydig and *Polyphemus pediculus* L. were also important prey for Arctic charr in Fjellfrøsvatn and Takvatn, whereas in Pulmankijärvi *Mysis* spp. opossum shrimps were abundant both in the zooplankton samples and in Arctic charr stomach contents. In essence, Arctic charr occupied a higher trophic position and showed a predominantly piscivorous diet in multispecies lakes where planktivorous prey fishes were available (Figs.3).

## Discussion

We found clear differences in the function (littoral vs. pelagic energy sources) and structure (number of trophic levels) of food webs between our 17 subarctic study lakes. The top predator Arctic charr shifted from littoral to more pelagic food resources with increasing lake size. This illustrates that, even though littoral benthic production typically dominates in small oligotrophic high-latitude lakes (cf. Sierszen et al. 2003; Karlsson and Byström 2005; Ask et al. 2009), top predators in larger high-latitude lakes shift to gain much of their energy from pelagic sources, derived from phytoplankton production. In multispecies fish communities, top predators have a higher trophic position due to piscivory on pelagic prey fishes. Hence, lake morphometry (particularly lake area) and fish species richness largely regulate the energy flow pathways and food-chain length in high-latitude lakes.

Littoral benthic algae have been reported to dominate primary production (Vadeboncoeur et al. 2003, 2008; Ask et al. 2009) and to act as the main energy source for top predators in small, oligotrophic, clear-water lakes (Karlsson and Byström 2005; Solomon et al. 2011; Eloranta et al. 2013b). In those ecosystems, the low nutrient concentrations in the water column limit pelagic phytoplankton production, while the clear water promotes production by benthic algae that can also access nutrients from the sediment. However, the relative contribution of littoral and pelagic production for whole ecosystem metabolism can be highly spatially and temporally variable within a lake (Sadro et al. 2011; Althouse et al. 2014; Hayden et al. 2014b). Moreover, in conjunction with previous stable isotope data from nine small subarctic lakes (Karlsson and Byström 2005), our results demonstrate that the predominant energy flow to top predators in oligotrophic high-latitude lakes changes fundamentally from littoral to pelagic with increasing lake size. Large lakes typically have longer open-water seasons and more extensive pelagic areas than small lakes, which promotes pelagic phytoplankton and zooplankton production (Wetzel 2001) and thus also facilitates energy flow to planktivorous and piscivorous fishes. Vadeboncoeur et al. (2008) found that the littoral benthic proportion of whole-lake primary production decreased with increasing depth ratio, light attenuation coefficient and trophic status. However, our study lakes are all oligotrophic (or slightly mesotrophic) and have clear water, and thus, the observed negative relationship between Arctic charr littoral reliance and lake size was not related to lake trophic state or water color. In contrast to our study, Vander Zanden et al. (2011) did not find significant relationships between lake-specific mean littoral reliance and morphometric or limnological variables in a survey comprising 546 fish populations across 75 lakes. The independence of fish littoral reliance from lake morphometry in their study could be due to a calculation of average mean littoral reliance across all fish species for each lake. Hence, their estimates of lake-specific mean littoral reliance for larger lake ecosystems likely include several littoral-dwelling fish species and do not only represent the predominant energy flow pathway supporting top predators. In contrast, our study focused on the resource use of a single generalist top predator species Arctic charr and thus better reflects differences in predominant energy sources along a lake-size gradient.

**Figure 4:**
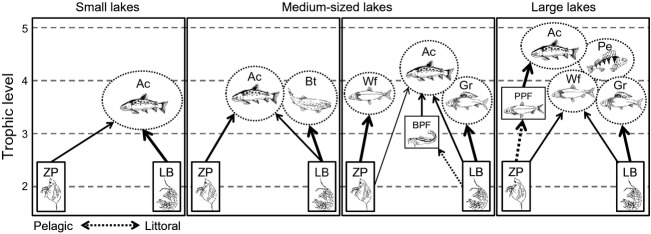
Schematic illustration of the trophic niche of Arctic charr (Ac) in high-latitude lake food webs with variable fish communities. Arctic charr mainly consume littoral benthic macro-invertebrates (LB) in small lakes, but shift to feed more on pelagic zooplankton (ZP) in medium-sized lakes if the littoral resources are dominated by brown trout (Bt). Alternatively, Arctic charr can prey upon benthic macro-invertebrates and benthivorous prey fish (BPF) such as minnow and small burbot if coexisting with abundant planktivorous whitefish (Wf) and benthivorous grayling (Gr). In large lakes with multispecies fish communities, including grayling and perch (Pe) as typical littoral competitors, Arctic charr shift to a predominantly pelagic, piscivorous niche by feeding on small planktivorous coregonid prey fishes (PPF). The boxes and ellipses indicate the putative food sources and the trophic niches of sympatric fish species, respectively, while the arrows indicate the trophic links of different strengths.

Both stable isotopes and stomach contents indicated that the contribution of littoral benthic prey in Arctic charr diet decreased with increasing lake size. This pattern may result from limited benthic algal production and concomitant low availability of benthic prey in large lakes. There, the pelagic niche of Arctic charr is likely supported by the availability of energetically profitable planktivorous prey fish, such as small pelagic whitefish and vendace, but may also be promoted by intense competition for littoral habitat and prey resources. Arctic charr may reduce competitive interactions with littoral fish species, including brown trout, whitefish, perch, and grayling, by shifting to a planktivorous or piscivorous diet in the pelagic or profundal habitat (Kahilainen and Lehtonen 2002; Eloranta et al. 2011, 2013a). In our study lakes, Arctic charr shifted to a more planktivorous diet in lakes where other efficient planktivores were absent or present in limited numbers. In multispecies lakes, Arctic charr seemed to specialize in feeding on small planktivorous prey fish in the pelagic and profundal habitats, where sympatric littoral-dwelling fishes were rarely found. There seems to be a strong pressure for early piscivorous niche specialization by Arctic charr in multispecies fish communities, as the smallest piscivorous individuals observed in those lakes were only 130–150 mm in fork length. Hence, the existence of small planktivorous prey fishes in large high-latitude lakes seems to promote niche segregation (i.e., reduce resource competition) between Arctic charr and sympatric benthivorous fishes, but also to shift the main energy flow pathway supporting these top predators from the littoral benthic to the pelagic phytoplankton-based food-web compartment (Fig.4). Similarly, as observed in Pulmankijärvi, Arctic charr may also shift to a more pelagic niche when large pelagic crustaceans like *Mysis* spp. are available. Karlsson and Byström (2005) found no difference in Arctic charr littoral reliance between lakes where Arctic charr were the only fish species or coexisted with nine-spined stickleback. However, in their small study lakes, pelagic production is likely limited and Arctic charr mainly consumed small benthivorous nine-spined sticklebacks and not larger planktivorous nine-spined sticklebacks which could have provided a pelagic trophic link between zooplankton and top predators, as the planktivorous coregonids did in our multispecies study lakes.

Although recent studies have presented partially conflicting evidence about factors determining food-chain length in aquatic and terrestrial ecosystems, the factors most often highlighted include ecosystem size, productivity, and disturbance (e.g., Takimoto and Post 2013; Warfe et al. 2013). Post and Takimoto (2007) suggested three structural mechanisms that can underlie variation in food-chain length in ecosystems: the addition or removal of (1) a top predator or (2) an intermediate consumer, or (3) a change in the degree of trophic generalization. As the number of fish species often increases with lake size (Barbour and Brown 1974), the increase in food-chain length with lake size may thus result from the addition of an intermediate consumer (e.g., a prey fish species) to the food web and from the subsequent piscivorous specialization of top predators (Vander Zanden et al. 1999b; Post and Takimoto 2007). Our results support the idea that both the addition of an intermediate consumer and the associated reduction in the degree of trophic generalization by top predators can influence the food-chain length in oligotrophic, high-latitude lakes (Fig.4). The observed negative relationship between Arctic charr trophic position and lake relative depth is most likely associated with the relatively shallow nature of the largest (>20 km^2^) study lakes as well as of Datkujavri and Vuolit Spielgajavri (Table S1) where Arctic charr preyed to a great extent upon other fishes. However, the relative importance of lake morphometric characteristics (e.g., area and relative depth) and fish species richness on Arctic charr trophic position and food-chain length is difficult to distinguish because the number of fish species is highly correlated with lake size for our study lakes (Pearson: *r *=* *0.72, *P *<* *0.001) as reported previously (Barbour and Brown 1974; Nolby et al. 2015). Comparing energy flow and food-chain length across high-latitude lakes of different size but with single-species fish communities could resolve this issue in the future.

Our study demonstrates the high potential of Arctic charr to alter their trophic niche and thus reflect fundamental differences in food-web structure and function (i.e., littoral vs. pelagic energy mobilization) in high-latitude lakes (Fig.4). The high niche plasticity of Arctic charr may not only reduce competitive interactions between sympatric fish species (e.g., Corrigan et al. 2011; Eloranta et al. 2011, 2013a; Woods et al. 2013), but also reduce consumer–resource oscillations and thereby increase the stability of food webs in high-latitude lakes (Rooney et al. 2006). For instance, the rapid behavioral responses of Arctic charr to seasonal fluctuations in benthic and pelagic production, including a temporary shift to predominantly zooplanktivorous diet in the late open-water season when littoral prey resources are scarce (Eloranta et al. 2013b; Hayden et al. 2014b), likely increase the stability of benthic and pelagic food-web compartments, also referred to as slow and fast energy channels, respectively (Rooney et al. 2006; Rooney and McCann 2012). Our study further supports the concept that generalist top predators can have a fundamental role in coupling littoral and pelagic habitats and food-web compartments in lake ecosystems (Schindler and Scheuerell 2002; Vander Zanden and Vadeboncoeur 2002). Across all 17 study lakes, average Arctic charr reliance on littoral and pelagic energy (carbon) sources was equal, consistent with the results of Hecky and Hesslein (1995) for littoral reliance of top predators in temperate and Arctic lakes. Habitat and food-web coupling by generalist top predators can be particularly strong in small ecosystems (Schindler and Scheuerell 2002; McCann et al. 2005), but is more limited in large lakes where increased heterogeneity and refuges may increase the density of prey fishes and thus promote trophic specialization (Post et al. 2000). In some high-latitude lakes, strong inter- and intraspecific resource competition may also reduce the potential of Arctic charr to exploit and integrate littoral and pelagic food-web compartments (Eloranta et al. 2013a).

Lakes are complex ecosystems in which mobile fish consumers play a particularly important role in predator–prey interactions, nutrient transfer between habitats, and in food-web structure and stability (Schindler and Scheuerell 2002; Rooney and McCann 2012). Hence, recognizing the factors determining the resource use by top predators is fundamental for evaluating the possible impacts of various disturbances on lake ecosystems, including climate-change-induced shifts in species composition (Jeppesen et al. 2010; Hayden et al. 2013) and in littoral and pelagic trophic pathways (Vadeboncoeur et al. 2003; Karlsson et al. 2009). Changes in littoral and pelagic production (bottom-up effects) and in foraging behavior of top predators (top-down effects) can both have strong impacts on food-web stability and ecosystem functioning in unproductive high-latitude lakes (Vadeboncoeur et al. 2003, 2005). Our results highlight how the function (i.e., littoral vs. pelagic energy flow) and structure (e.g., number of trophic levels) of food webs in high-latitude lakes are strongly associated with lake morphometry and fish community structure. Although littoral production typically dominates in small, oligotrophic, high-latitude lakes (Vadeboncoeur et al. 2003; Ask et al. 2009), our study shows that top predators rely substantially less on littoral production in larger high-latitude lakes, where planktivorous fishes provide a trophic link from pelagic zooplankton to the piscivorous Arctic charr. The existence of intermediate pelagic consumers as well as the strong interspecific competition for littoral resources in large lakes also promotes piscivory and concurrently increases trophic position of Arctic charr and lake food-chain length. In contrast, our results provide clear evidence that the littoral and pelagic food-web compartments are highly integrated in small- and medium-sized lakes where generalist top predators can exploit both benthic and pelagic resources.

## Data Accessibility

Data from the manuscript is publically available in the Dryad database (doi:10.5061/dryad.sc59f).

## Supporting Information

**Figure S1.** Location of study lakes in northern Finland and Norway.

**Figure S2.** Dietary proportions of benthic macro-invertebrates and fish regressed against lake area and fish species richness.

**Table S1.** Abiotic characteristics and fish community compositions in the 17 study lakes.

**Table S2.** Details of Arctic charr analyzed for stable isotopes and stomach contents.
