# Supplementary material for: Lake size and fish diversity determine resource use and trophic position of a top predator in high-latitude lakes
Source: Ecol Evol. 2015 Mar 23;5(8):1664–75. doi: 10.1002/ece3.1464 (PMC4409414; doi:10.1002/ece3.1464)
Supplement: Supplementary file 1 [file ece30005-1664-sd1.rtf]

Fig. S1. Location of study lakes in northern Finland and Norway.
