# Supplementary material for: Lake size and fish diversity determine resource use and trophic position of a top predator in high-latitude lakes
Source: Ecol Evol. 2015 Mar 23;5(8):1664–75. doi: 10.1002/ece3.1464 (PMC4409414; doi:10.1002/ece3.1464)
Supplement: Supplementary file 2 [file ece30005-1664-sd2.rtf]

Fig. S2. . Relative (arcsine square root transformed) proportions of (a) benthic macroinvertebrates and (b) fish in Arctic charr stomach contents modelled against lake surface area (ln km2) and fish species richness (ln n), respectively. The linear models are of the form: (a) , F1,15 = 11.4, R2 = 0.39, P = 0.004); (b) , F1,15 = 26.9, R2 = 0.62, P < 0.001).
