# Supplementary material for: Lake size and fish diversity determine resource use and trophic position of a top predator in high-latitude lakes
Source: Ecol Evol. 2015 Mar 23;5(8):1664–75. doi: 10.1002/ece3.1464 (PMC4409414; doi:10.1002/ece3.1464)
Supplement: Supplementary file 3 [file ece30005-1664-sd3.rtf]

Table S1. Abiotic characteristics and fish community compositions in the 17 study lakes. See Fig. S1 for full lake names.
Lake	Latitude (N)	Longitude (E)	Altitude (m a.s.l.)	Area (km2)	Littoral zone (%)	Zmax (m)	Zmean (m)	Zr (%)	D	Secchi depth (m)	Colour  (mg Pt l-1)	TN (ìg l-1)	TP (ìg l-1)	pH	AC % in catch	Fish species (n)	Fish species	
Tu	68°50'	27°07'	302	0.5	100	16	7	2.0	1.8	10	5	130	2	6.8	100	2	Ac,Bt	
Saa	69°05'	20°55'	679	0.7	53	24	5	2.5	1.6	11	5	100	4	7.2	100	2	Ac,Bt	
Vu	69°26'	23°33'	436	3.3	na	12	na	0.6	1.9	3	10	300	18	7.2	4	5	Ac,Bt,Wf,Gr,Bu	
Jo	69°15'	19°09'	91	3.4	na	48	15	2.3	1.7	9	6	170	6	8.0	46	3	Ac,Bt,3-sp	
Da	69°29'	23°26'	474	4.2	na	17	na	0.7	1.9	8	10	240	11	7.4	6	6	Ac,Bt,Wf,Gr,Mi,Bu	
Gæ	69°14'	23°00'	541	4.9	na	40	na	1.6	2.8	9	7	150	12	7.6	65	3	Ac,Wf,Bu	
Sag	69°11'	19°06'	91	5.1	na	80	na	3.1	1.6	9	9	140	5	7.9	54	3	Ac,Bt,3-sp	
Bi	69°33'	23°46'	381	5.4	na	52	na	2.0	1.6	8	10	150	3	7.1	17	7	Ac,Bt,Wf,Gr,Mi,Bu,Pi	
Fj	69°05'	19°20'	125	6.5	na	88	na	3.1	1.3	13	6	150	5	7.7	85	2	Ac,Bt	
Pu	69°58'	28°00'	12	12	13	36	20	0.9	2.0	4	30	180	4	7.1	2	9	Ac,Bt,3-sp,Wf,Gr,9-sp,As,Cb,Fl	
Li	69°00'	19°35'	102	13	na	92	31	2.3	1.5	11	11	240	6	7.4	44	6	Ac,Bt,Gr,Mi,Pi,Bu,As	
Ta	69°07'	19°05'	214	15	na	80	na	1.8	2.2	11	9	240	6	7.5	56	3	Ac,Bt,3-sp	
Uk	68°45'	27°28'	119	15	70	60	11	1.4	3.4	8	10	130	3	6.9	4	13	Ac,Bt,3-sp,Wf,Gr,Mi,Bu,Pi,Pe,Ve,9-sp,Ls,Lt	
Ra	68°45'	27°17'	132	22	43	46	14	0.9	4.5	7	10	100	3	7.1	3	11	Ac,Bt,3-sp,Wf,Gr,Mi,Bu,Pi,Pe,Ve,9-sp	
Ki	69°00	20°49'	473	37	29	57	19	0.8	2.4	10	5	120	4	7.2	2	8	Ac,Bt,Wf,Gr,Mi,Bu,Pi,Ab	
Mu	69°00'	26°50'	146	48	59	74	9	0.9	6.5	3	25	160	5	7.2	2	10	Ac,Bt,3-sp,Wf,Gr,Mi,Bu,Pi,Pe,9-sp	
In	68°58'	27°40'	118	1084	63	95	14	0.3	28.3	5	15	190	6	7.2	8	13	Ac,Bt,3-sp,Wf,Gr,Mi,Bu,Pi,Pe,Ve,9-sp,Ls,Lt	
Abbreviations: Littoral zone (%) = relative proportion (%) of littoral zone to whole lake area; Zmax, Zmean and Zr, = maximum, mean and relative depth, respectively; D = shoreline development; AC % in catch = proportion (%) of Arctic charr in total fish catch. Fish species: Ac = Arctic charr; Bt = brown trout;  3-sp = three-spined stickleback; Wf = whitefish; Gr = grayling; Mi = minnow; Bu = burbot; Pi = pike; Pe = perch; Ve = vendace; 9-sp = nine-spined stickleback; As = Atlantic salmon Salmo salar L.; Ls = land-locked salmon; Lt = lake trout; Ab = Alpine bullhead Cottus poecilopus Heckel; Cb = common bullhead; Fl = flounder Platichthys flesus L..
