# Supplementary material for: Lake size and fish diversity determine resource use and trophic position of a top predator in high-latitude lakes
Source: Ecol Evol. 2015 Mar 23;5(8):1664–75. doi: 10.1002/ece3.1464 (PMC4409414; doi:10.1002/ece3.1464)
Supplement: Supplementary file 4 [file ece30005-1664-sd4.rtf]

Table S2. Sampling years, number of samples (n) and fork length (FL) of Arctic charr analysed for stable isotopes (SIA) and stomach contents (SCA), and the estimated Arctic charr littoral reliance (LRcharr), trophic position (TPcharr) and stomach fullness. The values are means ± SD and ranges except LRcharr which is presented as means ± 95 % Bayesian credibility intervals (range indicates minimum and maximum extremes of the estimated LRcharr). The relative proportions (%) of empty stomachs analysed for SCA are also shown. See Fig. S1 for full lake names.
		SIA	FL (mm)	LRcharr (%)	TPcharr		SCA	FL (mm)	Fullness (%)	
Lake	Year	n	Mean	Range	Mean	Range	Mean	Range		n	Mean	Range	Mean	Empty	
Tu	2006	13	217 ± 47	151–307	70 ± 15	55–85	3.2 ± 0.1	3.0–3.3		30	187 ± 45	150–312	38 ± 22	10	
Saa	2011	16	343 ± 110	165–473	58 ± 11	47–70	3.3 ± 0.2	2.8–3.6		20	353 ± 102	165–473	41 ± 19	5	
Vu	2008	22	268 ± 92	154–488	62 ± 6	56–69	3.9 ± 0.3	3.2–4.3		22	268 ± 92	154–488	20 ± 30	23	
Jo	2010	70	240 ± 46	150–380	61 ± 5	56–66	3.6 ± 0.4	3.2–4.8		71	239 ± 47	150–380	25 ± 27	32	
Da	2009	12	375 ± 75	223–493	50 ± 8	43–58	4.6 ± 0.3	3.9–4.9		12	375 ± 75	223–493	40 ± 46	42	
Gæ	2009	89	243 ± 39	152–327	45 ± 4	42–49	3.2 ± 0.2	2.9–3.8		89	243 ± 39	152–327	51 ± 28	6	
Sag	2010	63	249 ± 61	150–340	35 ± 4	31–40	3.4 ± 0.3	3.0–4.5		73	237 ± 64	150–340	35 ± 28	19	
Bi	2008	35	243 ± 47	160–343	82 ± 8	74–90	3.4 ± 0.3	2.5–4.3		36	241 ± 47	160–343	21 ± 30	42	
Fj	2010	89	259 ± 54	152–415	62 ± 3	58–65	3.5 ± 0.3	3.0–4.8		125	238 ± 60	150–415	35 ± 33	32	
Pu	2009	43	256 ± 38	163–330	42 ± 6	36–48	3.7 ± 0.2	2.6–4.0		43	257 ± 37	163–330	20 ± 18	9	
Li	2010	59	245 ± 59	150–412	35 ± 4	30–39	3.4 ± 0.1	3.2–3.8		79	247 ± 52	150–412	30 ± 26	22	
Ta	2010	72	237 ± 59	150–435	64 ± 5	59–70	3.2 ± 0.3	2.6–4.1		72	237 ± 59	150–435	44 ± 32	15	
Uk	2010	21	270 ± 108	170–610	37 ± 7	31–43	4.0 ± 0.3	3.6–4.4		21	275 ± 110	173–621	36 ± 28	10	
Ra	2005	63	315 ± 88	151–476	32 ± 3	29–35	4.0 ± 0.2	3.1–4.3		63	320 ± 90	153–484	48 ± 28	5	
Ki	2005, 2006	50	451 ± 120	182–685	36 ± 3	33–38	5.1 ± 0.2	4.0–5.6		74	432 ± 123	159–698	27 ± 29	38	
Mu	2006, 2011	20	403 ± 104	157–573	45 ± 7	39–51	4.4 ± 0.2	4.1–4.8		177	374 ± 73	150–584	30 ± 33	37	
In	2009, 2010	67	377 ± 118	150–650	30 ± 5	25–35	4.4 ± 0.2	3.6–4.8		68	380 ± 123	151–663	15 ± 24	53	
